# Fowl Typhoid Outbreak on a Commercial Turkey Farm in Croatia

**DOI:** 10.3390/microorganisms12010165

**Published:** 2024-01-13

**Authors:** Liča Lozica, Siniša Faraguna, Branka Artuković, Željko Gottstein

**Affiliations:** 1 Department of Poultry Diseases with Clinic, Faculty of Veterinary Medicine, University of Zagreb, Heinzelova 55, 10000 Zagreb, Croatia; llozica@vef.unizg.hr; 2 Department of Pathophysiology, Faculty of Veterinary Medicine, University of Zagreb, Heinzelova 55, 10000 Zagreb, Croatia; sfaraguna@vef.unizg.hr; 3 Department of Veterinary Pathology, Faculty of Veterinary Medicine, University of Zagreb, Heinzelova 55, 10000 Zagreb, Croatia; abranka@vef.unizg.hr

**Keywords:** *Salmonella enterica*, Gallinarum, fowl typhoid, turkey, necropsy, histopathology, PCR, *flh*B

## Abstract

Fowl typhoid is a septicemic disease caused by *Salmonella enterica* subsp. *enterica* serovar Gallinarum biovar Gallinarum. It is a host-specific disease primarily affecting chickens and turkeys, although it has been reported in various animal species and sporadically in humans. Here, we present a case of a fowl typhoid outbreak on a turkey poult farm where the source of infection was the hatchery. The birds started showing symptoms of growth retardation at 21 days of age, after which the mortality rates gradually started to increase. Post mortem examination revealed that the main lesions were granulomatous proliferations in the small intestines. The results of the histopathological examination indicate that the severity of the infection was alleviated by the application of phytogenic mixtures and probiotics as a supportive treatment, even though the affected flock was eventually culled at 60 days of age. The farmer was advised to apply more strict biosecurity measures to prevent the spread of the disease on the farm and try to eradicate the pathogen from the barn. Since the outbreak, there have been no recurrent infections.

## 1. Introduction

*Salmonella enterica* subsp. *enterica* serovar Gallinarum (*S.* Gallinarum) is a host-specific, bacterial pathogen causing severe systemic infections in poultry [1,2,3]. Although *Salmonella enterica* serovars are present in a wide range of different environmental settings and hosts, *S.* Gallinarum is adapted to poultry and primarily affects chickens and turkeys [2,4,5]. Fowl typhoid and pullorum disease are septicemic diseases of poultry caused by biovars Gallinarum and Pullorum, respectively [6]. Both have a low incidence in most developed countries [5]. According to the meta-analysis for spatial distribution reported by Zhou and colleagues [5], the overall prevalence of *S.* serovar Gallinarum is the highest in Asia, specifically in eastern China. Interestingly, while Europe has the lowest prevalence of the biovar (bv.) Pullorum, it also has the highest prevalence of the biovar Gallinarum. However, the incidence of both diseases is probably underestimated because the majority of outbreaks are detected in backyard flocks [7] and often remain unreported, especially in developing countries.

*Salmonella* Gallinarum infections are considered diseases of chicks and poults, although fowl typhoid more often occurs in growing and adult birds, where it can cause mortality up to 90% [2,4]. The infected birds show weakness, anorexia, poor growth, and diarrhea [1,2]. The infection can be acute or chronic, so morbidity and mortality rates vary depending on the age and genetics of the birds, nutrition, zoohygienic conditions, husbandry system, ongoing co-infections, and entry of the pathogen [2]. Both biovars can be transmitted vertically and horizontally. However, the horizontal route through contaminated feed, water, litter, egg eating, cannibalism of the infected birds, farm equipment, wild birds, insects, or rodents, is considered more important for *S.* bv. Gallinarum [1,7,8,9]. Depending on the route of infection, the symptoms can be observed shortly after hatching [2]. Most commonly, the first clinical symptoms are seen after 2–3 weeks [2].

In this study, we present a case of a fowl typhoid outbreak on a commercial turkey farm in Croatia. The birds in the affected flock started showing symptoms approximately three weeks after hatching. Due to the severity of the infection and major losses to the farm, the birds were culled.

## 2. Materials and Methods

### 2.1. Case History

**Figure 1:**
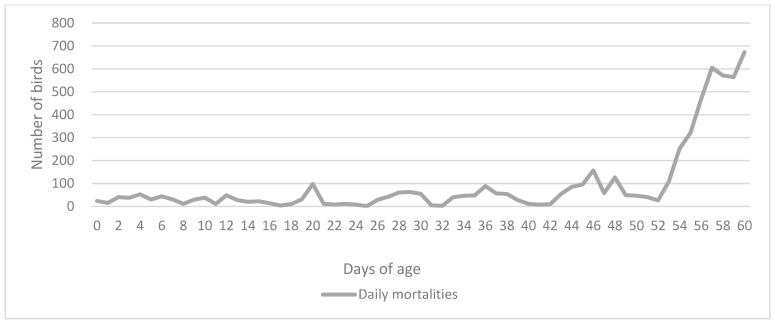
Daily mortality rates of the affected flock until the 60th day when the remaining sick birds were culled.

Slow-growing hybrid turkey poults suspected to have fowl typhoid were submitted to the Department of Poultry Diseases with Clinic (Faculty of Veterinary Medicine, University of Zagreb) for clinical and pathomorphological examination. Before that, samples of the transport cardboard boxes and birds that died during transport to the farm were collected and tested by a referral lab using the ISO method 6579-1 [10]. All samples tested negative for *Salmonella* spp. At the beginning of the rearing period, the farmer was informed about a *Salmonella* Gallinarum outbreak on another farm that supplies birds from the same hatchery and preventively submitted six 6-day-old poults for examination. At the time, the birds did not show any signs of disease. The submitted birds were euthanized and pathomorphologically examined. Internal organs, feces, and transport cardboard boxes were sampled again and tested using standard bacteriological examination (see below), and all were negative for *Salmonella*. Four out of six birds had yolk sac infections caused by *E. coli*. After three weeks, the birds started having occasional diarrhea. The flock showed growth retardation and significantly increased mortality rates. For the second sampling, two deceased and four live poults were examined, while for the third sampling, three deceased poults were examined (Table 1). The remaining birds in the flock were culled on the 60th day due to major economic losses and significant welfare issues (Figure 1).

The farm has a floor production system in eight detached poultry houses, which are set in the same location. The affected flock consisted of 10,160 poults held in one poultry house. The immunoprophylaxis program includes regular vaccination against Newcastle disease (ND) and turkey rhinotracheitis (TRT) by coarse spray in the hatchery, and revaccination for ND through drinking water at 35 days of age [11,12]. The birds are given supplements including probiotics, organic acids, essential oils, and phytogenic mixtures based on garlic, oregano, and thyme to enhance their overall performance and health. In the event of less severe disease symptoms, the birds on the farm are given alternative antimicrobials through feed or drinking water.

### 2.2. Clinical and Pathomorphological Examination and Sampling

After the clinical examination, live birds were euthanized and pathomorphologically examined. Altered organs at a macroscopic level including liver, pancreas, spleen, intestine, lungs, and bone marrow were sampled for further examination. Sampling involved taking swabs for bacteriological examination, collecting tissue samples for histopathological and molecular examination, taking intestine samples for coprological examination, and preparation of liver and bone marrow smears for cytological analysis.

### 2.3. Detection and Identification of Bacteria

After the examination and sampling of the poults, swabs (Copan, Brescia, Italy) taken from the liver, pancreas, spleen, intestine, lungs, and bone marrow were plated directly on MacConkey agar (Oxoid, Basingstoke, UK), Brilliant Green agar (Oxoid, Basingstoke, UK), UTI Brilliance Clarity chromogenic agar (Oxoid, Basingstoke, UK), and Columbia agar (Rapid Labs, Colchester, UK) enriched with 5% sheep blood (Biognost, Zagreb, Croatia). All plates were incubated aerobically at 37 °C for 24 h, while blood agar plates were additionally incubated anaerobically using AnaeroGen sachets (Oxoid, Basingstoke, UK) at 37 °C for 48 h. Identification was based on the morphological and biochemical characteristics of the bacterial colonies, molecular analysis, and serotyping using *Salmonella* antisera according to the White–Kauffmann–Le Minor scheme [13]. Serotyping was performed by the national accredited laboratory according to the standardized method EN/ISO 6579-1:2017/A1:2020 [10]. Determination of the biovar was performed based on the results of the ornithine decarboxylation test. The test was performed using Decarboxylase Moeller Base broth (Biolife, Milan, Italy) and L-Ornithine monohydrochloride (Merck KGaA, Darmstadt, Germany) according to the manufacturer’s instructions.

### 2.4. Antimicrobial Susceptibility Testing

The antimicrobial susceptibility of 15 randomly selected *S.* bv. Gallinarum strains, isolated from 7 different birds, was tested using a disk diffusion assay on Müller Hinton agar (Oxoid, Basingstoke, UK). The strains were tested against antibiotics which are frequently used on Croatian poultry farms. The inocula were prepared by suspending the bacterial colonies in sterile 0.9% saline solution adjusted to a turbidity of 0.5 McFarland units. Testing was performed with the following antibiotic discs: amoxicillin (10 μg), doxycycline (30 μg), enrofloxacin (5 μg), florfenicol (30 μg), norfloxacin (30 μg), and trimethoprim/sulfamethoxazole (25 μg) (Oxoid, Basingstoke, UK; Mast Group Ltd., Merseyside, UK). The inhibition zone diameters were interpreted according to the European Committee on Antimicrobial Susceptibility Testing (EUCAST) guidelines v13.0.

### 2.5. Molecular Analyses

Identification of *Salmonella* was confirmed using standard polymerase chain reaction (PCR) as described by Xiong et al. [14]. The DNA was extracted using the boiling method and Chelex 100 (BioRad, Hercules, CA, USA) according to the manufacturer’s instructions. The PCR products were visualized using electrophoresis on 1% agarose gel (Sigma-Aldrich, Co., St. Louis, MO, USA), stained using Midori Green Advance (Nippon Genetics Europe GmbH, Düren, Germany) in 1x TAE buffer (Promega, Madison, WI, USA).

### 2.6. Histopathology

Representative tissue samples of the intestines, pancreas, spleen, and liver were fixed in 10% neutral buffered formalin (Biognost, Zagreb, Croatia), dehydrated, embedded into paraffin, cut to a thickness of 5 µm, and stained with hematoxylin and eosin (HE) (Epredia, Breda, the Netherlands) for routine histopathological examination. Differential staining including Giemsa (Biognost, Zagreb, Croatia), Gram (Merck KGaA, Darmstadt, Germany) and Periodic acid-Schiff (PAS) (Merck KGaA, Darmstadt, Germany) was performed to detect bacteria, fungi, or parasites.

### 2.7. Cytology

Two imprints of the liver and three smears of the bone marrow were made aseptically. All submitted glass slides were air-dried for a few hours and stained using May–Grünwald Giemsa (MGG) (Biognost, Zagreb, Croatia). Slides were fully dried and the presence of intracellular bacteria was examined using a light microscope with an immersion oil.

### 2.8. Coprological Examination

Whole intestines were submitted for coprological examination using the flotation method in saturated sodium chloride solution, to exclude coccidiosis.

## 3. Results

### 3.1. Clinical and Pathomorphological Findings

**Figure 2:**
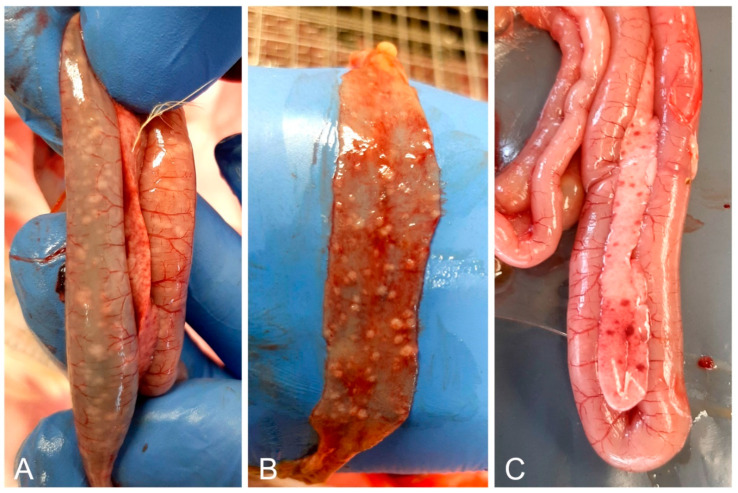
Most noticeable necropsy findings included white nodules on the duodenal (**A**) and ileal (**B**) mucosa, and petechial hemorrhages in the pancreas (**A**,**C**).

The submitted 28-day-old live birds were lethargic, stunted, and had diarrhea. All birds had the same lesions but these were less severe in the euthanized birds. Lesions included pneumonia, splenomegaly, petechial hemorrhages in the pancreas and under the gizzard cuticle, and granulomatous white proliferations in the liver and intestinal mucosa (Figure 2). The nodules in the intestines were prominent above the mucosa, with a radius of 2–4 mm. In some birds, the nodules were present only in parts of the intestine, most frequently in the duodenum or ileum. The 58-day-old birds had the same symptoms and lesions, but the birds were substantially more stunted for their age.

### 3.2. Laboratory Findings

**Figure 3:**
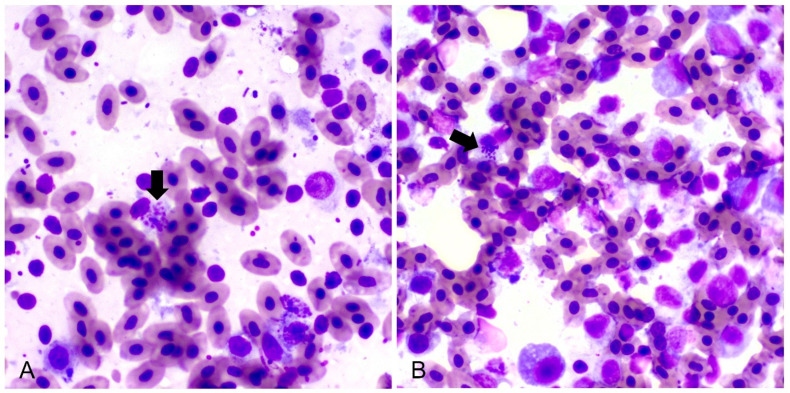
Impression smears of the liver (**A**) and bone marrow (**B**) showing intracellular rod-shaped bacteria (black arrows) (May–Grünwald Giemsa stain, 100×).

*Salmonella* was isolated from all swab samples taken during the second and third sampling. All samples were negative for clostridia. All tested DNA samples were positive for *Salmonella enterica* subsp. *enterica* serovar Gallinarum according to the serotypization and PCR analysis. The ornithine decarboxylation test was negative for all bacterial samples, confirming the identification of biovar Gallinarum. The susceptibility testing showed that all *Salmonella* strains were resistant to doxycycline, enrofloxacin, and norfloxacin (Table 2). When comparing strains isolated from the first and second sampling, they showed mostly intermediate (6/7) and high (8/8) susceptibility to florfenicol, respectively (Supplementary Table S1), indicating that the strain that caused infection changed over time. Liver impressions consisted of hepatocytes that were distributed as cellular clusters, as well as single cells, variable amounts of peripheral blood, few neutrophils, lymphocytes, and macrophages. Multifocally, some hepatocytes contained variable amounts of intracellular rod-shaped bacteria within the cytoplasm (Figure 3A). Bone marrow smears showed slight myeloid and megakaryocytic hypoplasia. Multifocally in the cytoplasm of some mature cells, rod-shaped bacteria were found (Figure 3B). All intestinal samples were negative for coccidia.

### 3.3. Histopathological Examination

**Figure 4:**
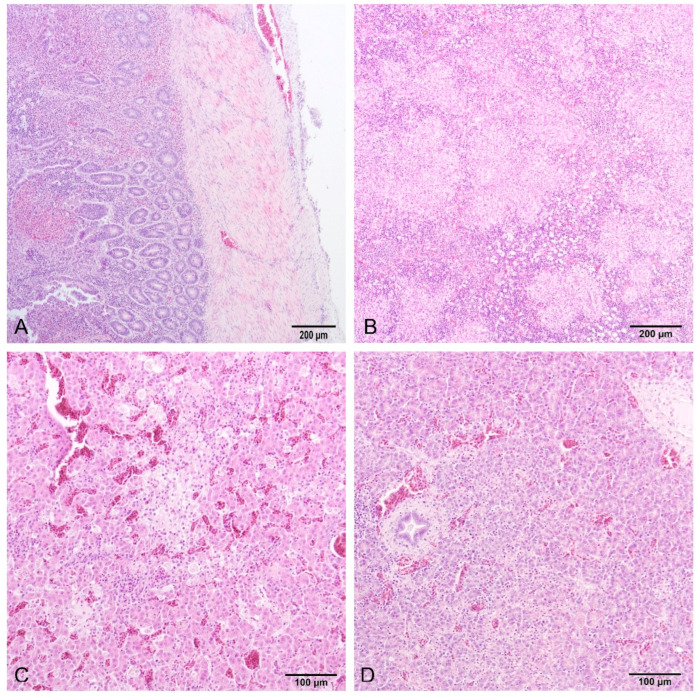
Small intestine (**A**) and spleen (**B**) of a deceased turkey poult, and liver (**C**) and pancreas (**D**) of a sacrificed turkey poult. (**A**) Fibrinonecrotic enteritis, and affected serosa (HE, 10×). (**B**) Necrohistiocytic splenitis, multifocal to coalescing, lymphocytic depletion in lymphoid follicles (HE, 10×). (**C**) Multifocal coagulative necrosis with inflammatory infiltrate, and congestia (HE, 20×). (**D**) Focal coagulative necrosis with inflammatory infiltrate, and congestia (HE, 20×).

Histology of the intestine revealed fibrinonecrotic enteritis (Figure 4A). Heterophils were predominant in the inflammatory infiltrate around the necrotic area, while accumulations of fibrin and cell detritus were detected in the intestinal serosa. The spleen was congested with multiple foci of necrosis with heterophils, macrophages (histocytes), and lymphocytes (Figure 4B). The liver had multifocal areas of necrosis comprising necrotic hepatocytes, heterophils, macrophages, and lymphocytes (Figure 4C). All findings were detected in samples both from the euthanized and deceased birds, except the pancreas. The pancreas samples of the deceased birds were congested, while in the pancreas of the euthanized birds, along with congestion, areas of coagulative necrosis and partial fibrosis of the tissue were found (Figure 4D). PAS and Giemsa staining were negative for fungi and parasites, respectively, while Gram staining showed mainly Gram-positive rod-shaped bacteria in the intestinal mucosa samples.

## 4. Discussion and Conclusions

The most obvious lesions we found were granulomas in the small intestine, which are considered a common finding in turkeys infected with *S.* bv. Gallinarum [4]. Lesions caused by fowl typhoid and pullorum disease are mostly similar in turkeys and chickens, except for ulcerations in the small intestine, which are uncommon in chickens [2]. In this case, the clinical symptoms that the farmer noticed were unspecific and relatively mild, possibly due to the continuous application of supportive treatment. The presence of Gram-positive rod-shaped bacteria in the intestines that were detected during histological examination was probably a result of the regular application of probiotics through feed. Also, the lack of Gram-negative rod detection, representing possible *Salmonella* colonization in the intestine samples, could indicate that application of the feed supplements was effective and acted according to the principle of competitive exclusion [15,16,17], which prolonged the period of intestinal colonization and probably alleviated the severity of the symptoms. The prolonged pathogenesis of the infection may have also been affected by the birds’ age, breed, feed, husbandry system, infection time, and dose, or specific pathogen characteristics [18,19]. Besides the differences in the immune response to the infection among different broiler breeds, slow-growing breeds may have a different intestinal morphology [19,20] that influences the bacterial colonization of the gut.

*S.* Gallinarum infection has been reported in humans, but the clinical cases are sporadic and considered a result of consumption of a high amount of contaminated food, so they are usually resolved without any antibiotic treatment [9,21]. On some poultry farms, antibiotics are still occasionally used to treat *S.* Gallinarum infections without veterinarians’ approval or supervision, regardless of the national program for the control of salmonellosis [22]. This approach is certainly contributing to the worldwide problem of increasing antimicrobial resistance (AMR) rates. Nevertheless, high AMR rates can also be detected on farms where no antibiotics have been used for a long period of time, due to the presence and circulation of resistant bacteria in the environment [23]. Bacteria can evolve through co-selection so that virulence and resistance genes are spread among the population of different commensal and pathogenic species. In the current case, antibiotic resistance to quinolones (enrofloxacin and norfloxacin) and tetracyclines (doxycycline) was detected, which are all commonly used to treat various bacterial infections in poultry production in Croatia. Exposure of *Salmonella* bv. Gallinarum to the residues of antibiotics or other bacterial species that were previously exposed to listed antibiotics could have led to the development of resistance in the current strains [23,24,25,26]. In the present study, a strong correlation was found between the presence of intracellular rod-shaped bacteria in the imprints of the liver, bone marrow, and small intestine, supporting the notion that the observed lesions were primarily a result of the bacterial infection.

The first fowl typhoid outbreak on a turkey farm usually causes the highest mortality, after which there can be intermittent recurrences and less severe losses [2]. In this case, the mortality rate during the outbreak reached 55.3% immediately before the culling of the birds, but there were no recurrences in the following three flocks on the farm. Since the source of the infection was the hatchery, the main management procedure on the affected farm was to prevent further spread of the disease and work on the elimination of *Salmonella* from the barn. The farmer was advised to apply more strict biosecurity measures, including more thorough cleaning and disinfection, and a prolonged downtime period. New flocks are regularly sampled, in addition to the official measures issued by the Commission Regulation (EU) (No 1190/2012) and the national Ministry of Agriculture [22], and there have been no recurrent outbreaks.

The presented case shows the importance of continuous and intensive monitoring of each flock, especially at the beginning of the production cycle, since hatcheries represent a common source of poultry pathogens. Thorough application of biosecurity measures acts as a preventive measure, as well as a continuous treatment method in contaminated poultry houses. In this case, the application of plant-based and probiotic feed supplements has proved to be effective in the prolongation of clinical manifestation and reducing the severity of the infection, which alleviated the effects of the poor biosecurity measures.

## Figures and Tables

**Table 1:** Timeline of the *Salmonella* Gallinarum outbreak on the farm.

Sampling	1st	2nd	3rd
Age of the birds (days)	6 d	28 d	58 d
Symptoms and lesions	no	yes	yes
*Salmonella* testing	negative	positive	positive

**Table 2:** Results of the antimicrobial susceptibility assay showing the number [%] of susceptible (S), intermediate (I), or resistant (R) strains to each tested antimicrobial agent.

	Antimicrobial Agent					
	AML ^a^ (10 μg)	DO (30 μg)	ENR (5 μg)	FFC (30 μg)	NOR (30 μg)	SXT (25 μg)
S	15 (100)	-	-	9 (60)	-	15 (100)
I	-	-	-	6 (40)	-	-
R	-	15 (100)	15 (100)	-	15 (100)	-

^a^ AML—amoxicillin, DO—doxycycline, ENR—enrofloxacin, FFC—florfenicol, NOR—norfloxacin, SXT—trimethoprim/sulfamethoxazole.

## Data Availability

Data is contained within the article or Supplementary Material.

## Supplementary Materials

The following supporting information can be downloaded at: https://www.mdpi.com/article/10.3390/microorganisms12010165/s1, Table S1: Results of the antimicrobial susceptibility assay and data on the origin of each tested strain.
